# Semantic intrusion errors differentiate between amnestic MCI who are plasma p-tau_217_+ from p-tau_217_- after adjusting for initial learning strength

**DOI:** 10.3389/fneur.2025.1613694

**Published:** 2025-07-22

**Authors:** Rosie E. Curiel Cid, David Vaillancourt, Alexandra Ortega, Elizabeth A. Crocco, Kirsten Crenshaw, Stephanie M. Remedios, Breton M. Asken, Melissa J. Armstrong, Idaly Velez Uribe, Wei-en Wang, Monica Rosselli, Malek Adjouadi, Michael Marsiske, Warren W. Barker, Steven DeKosky, Glenn Smith, Ranjan Duara, David A. Loewenstein

**Affiliations:** ^1^1Florida Alzheimer's Disease Research Center (ADRC), Miami, FL, United States; ^2^Center for Cognitive Neuroscience and Aging (CNSA), Department of Psychiatry and Behavioral Sciences, University of Miami Miller School of Medicine, Miami, FL, United States; ^3^Department of Applied Physiology and Kinesiology, Gainesville, FL, United States; ^4^Department of Clinical and Health Psychology, University of Florida, Gainesville, FL, United States; ^5^Department of Neurology, University of Florida College of Medicine, Gainesville, FL, United States; ^6^Wien Center for Alzheimer's Disease and Memory Disorders, Mount Sinai Medical Center, Miami Beach, FL, United States; ^7^Department of Psychology, Florida Atlantic University, Boca Raton, FL, United States; ^8^Center for Advanced Technology and Education, Florida International University, Miami, FL, United States; ^9^Department of Neurology and McKnight Brain Institute, University of Florida, Gainesville, FL, United States

**Keywords:** mild cognitive impairment, Alzheimer's disease, semantic interference, semantic intrusion errors, LASSI-L, plasma biomarkers, ptau217

## Abstract

**Background:**

Semantic intrusion errors (SIEs) are associated with mild cognitive impairment (MCI) due to Alzheimer's disease (AD). It is unknown whether accounting for maximum learning capacity still leads to an increase in SIEs when elevated plasma p-tau_217_, a biological indicator of underlying AD, is present.

**Methods:**

One hundred fifty-eight older adult participants completed the Loewenstein-Acevedo Scales for Semantic Interference and Learning (LASSI-L), a sensitive cognitive challenge test designed to elicit SIEs. Of these, 108 were clinically diagnosed with amnestic MCI (aMCI). Fifty-eight individuals met or exceeded a plasma p-tau_217_ positivity of >*0.5*5 pg/ml, while 50 individuals scored below this threshold.

**Results:**

After adjusting for demographic covariates and maximum learning capacity, the aMCI p-tau_217_+ group evidenced more SIEs compared to aMCI p-tau_217_- on the first (list B1; *p* = 0.035) and second trials of the competing list (list B2; *p* = 0.006). Biological predictors such as *ApoE* ε4 status, higher p-tau_217_, and older age were predictors of an elevated number of SIEs [list B2: *F* (3,104) = 10.92; *p* = 0.001; *R* = 0.489)].

**Conclusions:**

Unlike previous studies that used amyloid PET or other plasma biomarkers, individuals with aMCI p-tau_217_+ evidenced more SIEs, even after adjusting for their initial learning capacity, a covariate that has not been studied previously. These findings support that SIEs are more prevalent in the presence of underlying AD pathology and occur independent of learning deficits.

## 1 Introduction

Cognitive Challenge Tests such as the Loewenstein-Acevedo Scales for Semantic Interference and Learning (LASSI-L) present participants with two 15-item word lists that share semantically related targets. Identical semantic cues used during both encoding and retrieval trigger proactive semantic interference (PSI) effects. PSI occurs on the LASSI-L while trying to recall items from the second list. It represents a failure to learn new information in the face of recently learned *competing* information (1). It has been posited that PSI or failure to recover from PSI (fPSI), despite repeated presentations of the semantically competing word list, reflects a cognitive impairment that goes beyond a mere retrieval issue. This includes problems with source memory, inhibition, and, in the case of intrusion errors, the failure to self-monitor. These processes are thought to involve disruptions between medial temporal and prefrontal circuits (1).

PSI using the LASSI-L has been useful to detect prodromal AD (2) and has been associated with degeneration in AD prone regions (3, 4), which are predictive of disease progression (5). PSI has also been identified as an early cognitive deficit associated in other neurodegenerative conditions such as pre-manifest Huntington's disease (6, 7) and multiple sclerosis (8). Deficits in frPSI have been consistently observed in individuals with amnestic mild cognitive impairment (MCI) across diverse, multicultural cohorts in the United States and internationally (1, 4, 9–12). SIEs have consistently been observed in persons with underlying Alzheimer's disease (AD) pathology (1, 5, 12) and have shown a great deal of specificity to AD-related cognitive impairment. These errors primarily involve incorrectly recalling target items during PSI and frPSI trials of the LASSI-L (13). Individuals with amnestic MCI (aMCI) suspected to have an AD etiology, based on positive amyloid beta (Aβ) positron emission tomography (PET), demonstrate significantly more SIEs than aMCI older adults who are Aβ PET negative, despite both groups showing a clinically progressive disease course (5, 14, 15).

Blood tests for diagnosis of AD have been validated and found to have accuracy comparable to amyloid PET and cerebrospinal fluid (CSF) tests. This advancement offers exciting possibilities for larger-scale studies of AD-related cognitive phenotyping (16). One of the most promising blood-based biomarkers, plasma p-tau_217_, consistently demonstrates high sensitivity and specificity for Aβ PET positivity and tau PET results (17, 18). However, without AD-specific and culturally validated cognitive assessments, blood-based biomarkers may have limited applicability in primary care or community health settings.

Given the high concordance between plasma p-tau_217_ with neuropathological, CSF, and Aβ and tau PET biomarkers of AD, the objective of this study was to investigate the relationship between p-tau_217_ and AD-related cognitive markers such as frPSI and SIEs on the LASSI-L. A limitation of previous studies using the LASSI-L has been the failure to control for maximum learning capacity which may influence the occurrence of frPSI and SIEs. In this study, we addressed this previous limitation by using maximum learning capacity on the first target list of the LASSI-L. It was hypothesized that individuals with aMCI and higher p-tau_217_ levels, would exhibit an increased number of SIEs and a higher percentage of SIE impairments, based on established cut-off values.

## 2 Methods

### 2.1 Population

An ethnically diverse cohort of participants (>50% Hispanic/Latino) were enrolled in the 1Florida Alzheimer's Disease Research Center (ADRC), an Institutional Review Board-approved observational study. All participants underwent a comprehensive screening process and persons with major neurological, psychiatric, and systemic medical conditions known to affect cognition, including neurodegenerative disease, mood disorders, traumatic brain injury, and current substance abuse were excluded. We selected people diagnosed with aMCI (*n* = 98) who underwent an extensive evaluation inclusive of a clinical interview, medical history and physical examination, blood draw, brain magnetic resonance imaging (MRI), and a comprehensive neuropsychological battery. In addition, a collateral informant was interviewed using the Clinical Dementia Rating (CDR) scale (19, 20).

A bilingual trained psychometrician administered the standardized neuropsychological battery in the participant's dominant and preferred language (English or Spanish). The neuropsychological battery used to classify older adults by clinical syndrome included the Hopkins Verbal Learning Test-Revised (HVLT-R) (21) immediate and delayed memory scores, delayed recall on the National Alzheimer's Coordinating Center's (NACC) Uniform Data Set (UDS) story passages (22), the Controlled Oral Word Association Test: Category Fluency and Phonemic Fluency (23), Block Design subtest of the Wechsler Adult Intelligence Scale, Fourth Edition (WAIS-IV) (24), and the Trail Making Test (Parts A and B) (25). The LASSI-L was not part of the diagnostic battery.

An experienced clinician evaluated each participant while remaining blind to the results of neuropsychological tests. Similarly, the neuropsychologist interpreting the cognitive test results was blind to the clinical impressions prior to the consensus diagnosis. The diagnosis of aMCI was determined through an interdisciplinary consensus approach that integrated both clinical and neuropsychological data. The following diagnostic criteria were used for classifying aMCI: (1) subjective cognitive complaints reported by the participant and/or a collateral informant; (2) a CDR global score of 0.5, indicating no significant functional impairment suggestive of dementia or a major neurocognitive disorder as defined by DSM-5; and (3) impaired delayed recall (i.e., 1.5 standard deviations or greater below the mean, adjusted for age, education, and language) on either the HVLT-R or delayed paragraph recall (verbatim) from the NACC UDS. In addition to evidence of an amnestic problem, a score that was 1.5 SD (26) or more, below expected levels on non-memory measures, was also accepted for individuals with amnestic multi-domain MCI. Many participants in the amnestic MCI group exhibited impairments in non-memory domains; therefore, the statistical power to determine whether aMCI alone or impairment in non-memory domains influenced outcomes was limited.

### 2.2 Calculation of plasma p-tau_217_ and derivation of cut-offs

Venous blood was collected using 10 ml Purple Top tubes containing EDTA as an anticoagulant, mixed by inversion 10 times, and centrifuged at room temperature for 12 min at 1,200 rcf within 1 h of collection. Aliquots of 500 μl of plasma were then frozen and stored at −80°C. Prior to analysis, the samples were thawed (one freeze-thaw cycle) at room temperature, vortexed for 30 s, and placed on ice until centrifuging at 10,000 g for 5 min at 4°C. Duplicate samples were analyzed at the Quanterix Accelerator Lab blinded to all clinical and demographic data, using single molecule array (SIMOA) technology for p-tau_217_ (ALZpath; Quanterix, Billerica, MA). We only included samples with a coefficient of variation 20% or less. It is common practice in our laboratories and others worldwide to exclude p-tau_217_ values that have a duplicate covariation of >20%. This is because the reliability of the findings is questionable.

Internally derived cutoffs for “positive” plasma p-tau_217_ were based on correspondence with the visual read of an Aβ-PET scan contiguous with the blood draw from our 1Florida ADRC (*N* = 239). We performed receiver operating characteristic (ROC) curve analyses using amyloid PET as the gold standard, correlating it with plasma levels of p-tau_217_ measured by SIMOA (Alzpath). The area under the ROC curve was 0.91 (*p* < 0.001), indicating strong discriminative ability. To identify the optimal classification thresholds for p-tau_217_ (pg/ml), we applied Youden's index (27). Our analysis revealed that a p-tau_217_ cut-off of 0.55 pg/ml provided the best balance, yielding 89.0% sensitivity and 82.4% specificity (28). Recently in other cohorts, Ashton et al. (16) have suggested that cut-offs for plasma p-tau_217_ as high as >63 pg/ml are extremely suggestive of significant underlying AD pathology but that that plasma p-tau_217_ values of >0.55 likely represents abnormal accumulation of AD related proteins within the brain (16).

### 2.3 Loewenstein-Acevedo scales for semantic interference and learning (LASSI-L)

The LASSI-L cognitive stress test employs controlled learning during acquisition to maximize the storage of a list of to-be-remembered target words belonging to one of three semantic categories (fruits, articles of clothing, and musical instruments) (29). The LASSI-L is culturally fair and valid in either English or Spanish (2, 30).

During the administration of the LASSI-L, the examinee is asked to remember a list of 15 common words (list A) representing three semantically distinct categories over two learning trials to maximize storage and consolidation. Subsequently, a different list of semantically competing words is presented in the same manner as the first list. The second list (list B) introduced different target words, but the words across both lists shared the same semantic categories to elicit proactive semantic interference (PSI). Unlike traditional memory assessment paradigms, the individual's ability to recover from the effects of PSI (frPSI) is possible because of the repeated administration and cued recall of the competing word list (list B) (10, 29–31).

In the current study, since our focus was on different aspects of PSI and frPSI, we focused on: (a) correct responses during cued recall trials of list B (Cued B1 and Cued B2), as well as total semantic intrusion errors (SIEs) made on these trials. This version of the test takes 12 min or less to administer (32). In this study, as well as previous investigations, the vast majority (>90%) of intrusion errors consist of words from the competing word list (13). Semantic intrusion errors may also occur when an individual intrudes a word from the same semantic universe that is not a target word on either list A or list B.

### 2.4 Aβ PET imaging

PET computed tomography (CT) imaging, using a 3D Hoffmann brain phantom, established a standardized acquisition and reconstruction method. Participants were infused with [18-F] florbetaben 300 MBQ over a 3-min period. The scan occurred 70–90 min post-infusion for a duration of 20 min. All participants were scanned on a Siemens Biograph 16 PET/CT scanner operating in 3D mode (55 slices/frame, 3 mm slice thickness 128 × 128 matrix). The PET data were reconstructed into 128 × 128 × 63 (axial) matrices with voxel dimensions of 0.21 × 0.21 × 0.24 cm. Reconstruction was performed using manufacturer-supplied software and included corrections for attenuation, scatter, random coincidences, and dead time. Images for regional analyses were processed using Fourier analysis followed by direct Fourier reconstruction. Images were smoothed with a 3 mm Hann filter. Following reconstruction, image sets were inspected and, if necessary, corrected for inter-frame motion. Images were obtained from the top of the head to the top of the neck and CT data was employed for initial attenuation correction and image reconstruction in the sagittal, axial, and coronal planes.

The PET/CT images, including the outline of the skull, were co-registered linearly (i.e., trilinear interpolation) with 12 degrees of freedom, onto the volumetric MRI scan using a T1-weighted [magnetization-prepared rapid gradient-echo (MP-RAGE)] image (33). Region-of-interest boundaries were defined manually using the structural MRI for anatomical reference, and criteria that have been proven to provide highly reproducible outcomes (34). This registration process ensured that the PET/CT image had the same accurate segmentation and parcellation as the MRI scan. The PET/CT and MR reconstruction were performed using manufacturer-provided software. Registration between PET/CT and the MR using FSL's FLIRT (35) algorithm was employed. A Siemens Skyra 3 T MRI scanner (Siemens Medical Solutions, Erlangen, Germany) was used with MRI acquisition of 1 h. The 3D T1-weighted volumetric magnetization-prepared rapid gradient-echo sequence (MP-RAGE) consisted of 176 slices at slice thickness = 1 mm isotropic, FOV = 256 × 256, TR = 3.0 s, TE = 1.4 s, and flip angle = 9°. Over 80 percent of our Aβ PET scans utilized florbetaben as the primary tracer, with the rest of participants having had florbetapir scans. Thus, we employed the Centiloid method to create a common metric by which total uptake can be placed on the same scale for different Aβ tracers (36, 37).

### 2.5 Visual ratings of Aβ PET scans

All Aβ PET scans were interpreted by an experienced neuroradiologist who was blind to the cognitive and clinical diagnoses, using a methodology similar to that described by Loewenstein et al. (10) and Curiel Cid et al. (38). Using that methodology, an interrater reliability of 98% for amyloid visual reads was obtained. A final dichotomous amyloid positive (A+) vs. amyloid negative (A–) diagnosis was rendered. Visual reads of Aβ PET are considered the gold standard in the field (39).

### 2.6 Apolipoprotein E (*ApoE*) genotyping

*ApoE* genotyping was performed in Dr. Nilüfer Ertekin-Taner's laboratory at the Mayo Clinic in Jacksonville, FL, USA. *ApoE* ε2, ε3, and ε4 alleles were analyzed with predesigned TaqMan SNP Genotyping Assays for SNPs rs7412 and rs429358 (Thermo Fisher Scientific, Waltham, MA, USA) on the QuantStudio 7 Flex Real-Time PCR system (Applied Biosystems, Carlsbad, CA, USA). Individuals who were homozygous or heterozygous for the ε4 allele were classified as *ApoE* ε4 positive.

### 2.7 Statistical analyses

The first comparisons of demographic, biomarker, and LASSI-L indices were conducted using a series of one-way analyses of variance (ANOVA). When a statistically significant result was obtained with interval level data for the two study groups, (aMCI p-tau_217_+ vs. aMCI p-tau_217_-), Cohen's *d* was calculated to determine effect sizes. Dichotomous variables were analyzed by 2 × 2 Chi-square analyses with Yate's correction for discontinuity. In ANOVA analyses, we ran non-parametric Mann–Whitney *U* tests of ranks and conducted analyses using transformed scores which did not result in any changes in the obtained results.

Statistically significant covariates such as age, Mini Mental State Exam (MMSE) score, and strength of initial learning (measured by LASSI-L Cued A2) were then subsequently adjusted in ANCOVA models.

Finally, we determined the relationships biological measures such as sex, *ApoE* ε4 genotype, and p-tau_217_ status in the entire sample to predict the number of semantic intrusions utilizing multiple linear regression models using both simultaneous entry (each variable is evaluated adjusting for all other variables in the model), as well as step-wise approaches to determine effect size changes as more biological variables were entered in the model. The data did not have extreme outliers, and the data was sufficiently normally distributed to conduct proposed analyses.

## 3 Results

As depicted in Table 1, participants classified as aMCI p-tau_217_+ did not differ from those who were aMCI p-tau_217_- with regards to years education attained, sex, or Hispanic/Latino ethnicity. Participants who were aMCI p-tau_217_+ were older [*F* (1,107) = 4.10; *p* = 0.045], had lower MMSE scores [*F* (1,107) = 6.98; *p* = 0.009], and a higher percentage of Aβ positive PET scans (87 vs. 13%) [*X*^2^ (df = 1) = 44.01; *p* < 0.001]. In addition, those who were p-tau_217_+ were more often carriers of the *ApoE* ε4 allele (54.2 vs. 32.0%) [*X*^2^ (df = 1) = 4.57; *p* = 0.03]. Factors such as being Hispanic/Latino or language of evaluation had no effect on obtained results.

**Table 1:** Diagnostic groups demographic, LASSI-L indices and plasma biomarkers.

**Variable**	**aMCI p-tau_217_– (*n* = 50)**	**aMCI p-tau_217_+ (*n* = 58)**	***F*-or *X*^2^ value**	***p*-Value**	**Cohen's *d***
Age (range 56–93)	70.80 (SD = 7.8)	73.59 (SD = 7.3)	4.10	0.045	0.689
Education (range 6–22)	15.33 (SD = 3.6)	15.80 (SD = 3.1)	0.452	0.503	NA
MMSE score (range 23–30)	28.18 (SD = 1.7)	27.00 (SD = 2.2)	6.98	0.009	0.303
% Female	59.2%	47.5%	1.044	0.317	NA
% Hispanic/Latino	59.2%	50.8%	0.452	0.501	NA
% *ApoE* ε4 positive	32.0%	54.2%	4.57	0.033	NA
% Aβ PET positive	13.0%	87.0%	44.01	< 0.001	NA
p-tau_217_ (range = 0.180–3.15)	0.344 (SD = 0.103)	1.167 (SD = 0.570)	121.042	< 0.001	2.446
LASSI-L Cued A2 recall (range 6–15)	12.26 (SD = 2.2)	10.97 (SD = 2.4)	8.71	0.004	0.561
LASSI-L Cued B1 recall (range 6–15)	5.52 (SD = 2.7)	5.74 (SD = 2.4)	0.211	0.647	N/A
LASSI-L Cued B1 intrusions (range 0–13)	3.28 (SD = 3.0)	5.47 (SD = 3.5)	11.92	< 0.001	0.674
LASSI-L Cued B2 recall (range 3–15)	9.90 (SD = 2.5)	8.33 (SD = 2.4)	11.11	0.001	0.641
LASSI Cued B2 intrusions (range 0–13)	2.34 (SD = 2.0)	4.36 (SD = 2.9)	17.10	< 0.001	0.824

As it relates to LASSI-L performance, participants who were aMCI p-tau_217_+ had lower initial learning scores (Cued A2) [*F* (1,107) = 8.71; *p* = 0.004] and lower recall on the trial that measures frPSI (Cued B2 recall) [*F* (1,107) = 11.11; *p* = 0.001]. Participants who were aMCI p-tau_217_+ also had more SIEs on trials that measure PSI and frPSI (Cued B1 intrusions) [*F* (1,107) = 11.92; *p* < 0.001, *d* = 0.67]; and Cued B2 intrusions [*F* (1,107) = 17.10; *p* < 0.001, *d* = 0.82]. Cohen's *d* was calculated for the LASSI-L Cued B2 recall (*d* = 0.64); Cued B1 intrusions (*d* = 0.67) and Cued B2 intrusions (*d* = 0.82) and yielded moderate to large effect sizes.

Results were generally consistent after statistical adjustments for age, MMSE, and maximum learning scores (Cued A2) using a series of ANCOVA models evaluating SIEs on Cued B1 and Cued B2 (Table 2). Even after this covariate adjustment, aMCI p-tau_217_+ participants continued to evidence more SIEs on Cued B2 (*p* = 0.006) and Cued B1 (*p* = 0.035), albeit to a lesser extent. It should be noted in Table 2, mean values adjusted for covariates with their standard errors of measurement are depicted. While ANOVA and ANCOVA models are mathematically equivalent and specialized cases of linear regression, we conducted separate regression analyses that focused on biological predictors of SIEs independent of diagnostic status. Since sex as a biological variable was not found to be related to SIEs, we only included the three biological variables that were available to us (age, p-tau_217_ positivity, and ApoE4 positivity) as predictors of SIEs in the entire sample. When all variables were entered simultaneously in a linear regression model, each was statistically significant (Table 3). We also employed a stepwise regression approach which ordered predictors in the model resulting in a Total *R* of 0.489 (*R*^2^ = 0.240; Table 4).

**Table 2:** Plasma p-tau_217_ biomarker status and LASSI-L performance after adjustment for age, MMSE and maximum learning capacity on Cued A2.

**LASSI-L Variable**	**aMCI p-tau_217_– (*n* = 50)**	**aMCI p-tau_217_+ (*n* = 58)**	***F*-value**	***p*-Value**
LASSI-L Cued B1	5.18 (SE = 0.34)	6.04 (SE = 0.31)	3.31	0.072
LASSI-L Cued B1 Intrusions	3.73 (SE = 45)	5.08 (SE = 0.42)	4.56	0.035
LASSI-L Cued B2	9.38 (SE = 2.9)	8.78 (SE = 2.6)	2.28	0.134
LASSI-L Cued B2 Intrusions	2.69 (SE = 0.35)	4.06 (SE = 0.32)	7.87	0.006

These are mean values adjusted for covariates with their standard errors of measurement. After statistical adjustment, only SIES were statistically significant.

**Table 3:** Biological predictors of LASSI-L Cued B2 intrusions for entire sample (simultaneous entry).

**Predictor**	***B***	**SE**	**Standardized beta**	***t*-value**	**Significance**	**Lower bound 95% Confidence interval**	**Upper bound 95% Confidence interval**
ApoE 4 Allele	1.75	0.50	0.32	3.52	*p* < 0.001	0.76	2.7
Age	0.07	0.03	0.19	2.16	0.033	0.01	0.13
p-tau_217_	1.15	0.44	0.24	0.2.61	0.01	0.28	2.02

Each variable is adjusted for every other statistical variable in the model.

**Table 4:** Biological predictors of LASSI-L Cued B2 intrusions for entire sample (stepwise entry).

**Model**	***R***	**R Square**	**Adjusted R Square**	**Std. Error of the estimate**	**R square change**	***F* change**	**df1**	**df2**	**Sig F. change**
1	0.366^a^	0.134	0.126	2.541	0.134	16.371	1	106	< 0.001
2	0.453^b^	0.206	0.190	2.445	0.072	9.485	1	105	0.003
3	0.489^c^	0.240	0.218	2.404	0.034	4.645	1	104	0.033

^a^Predictors: (Constant), APOE Genotype.

^b^Predictors: (Constant), APOE Genotype, p-tau_217_.

^c^Predictors: (Constant), APOE Genotype, p-tau_217_, Age.

Note that R squared and eta square represent effect sizes.

## 4 Discussion

Individuals with clinically diagnosed MCI and underlying Alzheimer's disease pathology exhibit more semantic intrusion errors (SIEs) than those with MCI of similar severity due to other etiologies, such as multiple cerebral infarctions, neurological disorders, or psychiatric conditions (40, 41). SIEs and false recognition among aMCI patients may be related to AD pathology, as measured by Aβ deposition on PET or low CSF levels of Aβ (1-42) in aMCI (42, 43). Thomas et al. (44) found that intrusion errors on the Rey Auditory Verbal Learning Test at baseline could predict progression to MCI among cognitively unimpaired individuals studied longitudinally in the Alzheimer's Disease Neuroimaging Initiative (ADNI). Similarly, Crocco et al. (31) found that SIEs on the LASSI-L could predict those persons with aMCI who would progress to dementia. Loewenstein et al. (10) and Kitaigorodsky et al. (5) found that SIEs on a cognitive challenge test that measured proactive semantic interference (PSI) and the failure to recover from proactive semantic interference (frPSI) could differentiate between older adults with aMCI who were Aβ PET positive, from persons diagnosed with aMCI who were Aβ PET negative, as well as cognitively unimpaired individuals who were Aβ PET negative.

One of the unique aspects of the LASSI-L assessment paradigm is that it was designed to elicit semantic intrusion errors because the test is structured to maximize learning of two semantically competing word lists. While detection of SIEs is partially possible on other list learning test such as the California Verbal Learning Test, these tests may have a few items that overlap semantically between lists, but do not use a 1:1 semantic competing list, thus, they will fail to capture SIEs. Methods have been developed by Thomas et al. (44) that use neuropsychological process scores that have related word list intrusion errors in the Alzheimer's Disease Neuroimaging Initiative (ADNI) dataset where she found that these errors improved earlier identification of persons with subtle cognitive decline.

Longitudinal studies suggest that SIES may have predictive utility for determining transitions from PreMCI to MCI, and MCI to dementia as well as longitudinal changes in CDR sum of boxes. Zheng et al. (45) investigated the predictors of SIEs in a longitudinal path analysis involving 216 participants from the 1Florida ADRC. This analysis accounted for covariates such as sex, education, language of evaluation, and overall cognitive impairment. The results revealed that both Aβ PET positivity and reduced brain volumes in regions vulnerable to AD were directly associated with SIEs. Additionally, the APOE ε4 status influenced SIEs indirectly by affecting amyloid positivity. Age also had an indirect effect on SIEs through its relationship with Aβ positivity and brain volumes. Notably, global mental status did not significantly impact these findings. In a study conducted by Crocco et al. (31) found that SIEs are predictive of progression of cognitive impairment in persons diagnosed with aMCI or PreMCI (not cognitively normal but not meeting full criteria for MCI). Additional longitudinal studies are ongoing to evaluate whether elevated SIE rates predict future progression to biologically confirmed AD/ADRD.

This study is among the first to link an AD-relevant cognitive marker (SIEs on LASSI-L's PSI and frPSI measures) to the promising plasma-based AD pathology marker, p-tau_217_. This association persisted after adjusting for initial learning capacity. It is likely that these deficits likely extend beyond source memory and involve deficits in self-monitoring (1). Semantic inhibition and self-monitoring difficulties may significantly contribute to semantic intrusions. Chasles et al. (46) found that impaired deep semantic processing deficits, beyond source memory and general inhibition, exacerbated semantic interference on the LASSI-L compared to a phonemic processing test.

Previous studies using path analytic models have elegantly shown that among those with both Aβ PET positivity and neurodegeneration in AD prone regions, *ApoE* ε4 genotype has an indirect effect on SIEs through Aβ PET positivity (45). These previous studies were limited in that they did not examine SIEs in relation to initial learning capacity [measured by maximum recall score (Cued A2) after two list A trials]. The strength of initial learning appears to attenuate Cued B1 SIEs but does not have nearly the effect on the attenuation of SIEs that are elicited on Cued B2, even though two consecutive learning trials of list B had occurred at that point. It might be argued that initial strength of encoding and recall of the original list A targets might have created the opportunity for greater proactive semantic interference when semantically competing list B items were presented using identical category retrieval cues (fruits, musical instruments, and articles of clothing). However, in this study, the degree of initial learning of list A targets had less of an effect on SIEs on Cued B2 among aMCI p-tau_217_+ vs. aMCI p-tau_217_-. This indicates that SIEs generated during a cognitive challenge measured by the frPSI most likely represented a continuous breakdown in self-monitoring and response inhibition in the face of semantically competing information, despite continued opportunities to learn. We postulate that the LASSI-L elicits a far greater number of semantic intrusions than other list learning tasks given that both the A list and B list contain 15 targets that have identical semantic cues used during both acquisition and retrieval. Presenting identical semantic cues on list B Cued B1 and Cued B2 retrieval trials likely enhances the semantic interference effect (10, 47) that occurs during activation of both semantic and episodic memory circuits that have been conceptualized as interdependent (48) and affected during the earliest stages of prodromal AD.

The mechanisms underlying frPSI are being more actively explored. Recently, Valles-Salgado et al. (49) reported that low correct responses on tests sensitive to frPSI on the LASSI-L are associated with ^18^F fluorodeoxyglucose (FDG) PET abnormalities in AD-relevant regions, including the medial temporal lobes, precuneus, and posterior cingulate. Rolls (50) postulated that there are connections between the limbic regions and cortical systems, with the posterior cingulate cortex being involved in memory due to its connections with the hippocampal memory system. With regards to SIEs, Sánchez et al. (12) found that the only LASSI-L measures that distinguished between middle-aged asymptomatic children of persons diagnosed with late-onset AD from controls were SIEs on Cued B2 of the LASSI-L. Moreover, the number of such intrusion errors were related to corticolimbic disconnection on fMRI. Taken together, these findings have led to the conclusion that SIEs, particularly as they occur during tests of frPSI, reflect deficits in self-monitoring and the inability to inhibit previously learned responses (1).

This study enhances our understanding of cognitive breakdowns and their links to promising plasma biomarkers of AD. We found that Cued B2 SIEs were predicted by a multivariate combination of ApoE ε4, p-tau_217_ positivity, and age, with explained variance in Cued B2 SIEs surpassing that previously reported for p-tau181 (1).

Strengths of the current investigation include a comprehensively evaluated and broadly representative sample of older adults with plasma p-tau_217_ levels and *ApoE* ε4 genotyping, enabling comparison on tests measuring important cognitive markers relevant to prodromal AD namely PSI, frPSI, and SIEs. Additionally, there were no significant demographic differences between the p-tau_217_+ and p-tau_217_- groups on important variables such as years of education, sex, or Hispanic/Latino ethnicity. Another strength was statistical adjustment for baseline differences with regards to age and MMSE scores in aMCI participants across the p-tau_217_+ and p-tau_217_- groups.

A potential limitation of the study is the insufficient number of cognitively normal and preMCI participants (cognitively impaired-not MCI) who are p-tau_217_ positive, limiting meaningful statistical comparisons with the current aMCI sample. Although ApoE ε4, p-tau_217_ levels, and age were predictive of Cued B2 SIEs, a significant portion of the variability in SIEs remained unexplained by the regression models, which represents a potential limitation of the current study. This unexplained variance may stem, in part, from differences in cognitive reserve in the presence of brain pathology. Furthermore, beyond the scope of this investigation, emerging research suggests that factors such as neuroinflammation and co-pathologies may mediate or moderate the relationship between p-tau217 and SIEs. Future studies with larger sample sizes and longitudinal follow-up are needed to better understand the predictive value of plasma biomarkers and deficits in self-monitoring and semantic inhibitory control (as reflected by SIEs) in relation to clinical progression over time (51, 52). Given the brief administration time of the LASSI-L, SIEs could serve as a useful tool to identify individuals who may require more detailed assessments for clinical trial inclusion. As AD/ADRD research advances toward blood-based biomarker validation for clinical use, the interpretive value of these biomarkers will be enhanced if they align with AD-specific cognitive deficits, such as SIEs, which are easily measurable errors indicative of clinically relevant decline.

While the study sample is ethnically diverse, participants were recruited from a single research center in South Florida, which may limit applicability to more culturally or geographically heterogeneous populations. It is also important to note that although bilingual assessment was conducted in participants' preferred language (Spanish or English) by fluently speaking and culturally trained clinicians, subtle culturally based linguistic deviations may influence performance on verbal memory tasks, specifically in semantic intrusion errors, and differ in other bilingual or monolingual populations. Statistical modeling was standard for a study of this type but focused on individuals with amnestic MCI. As noted in the Discussion Section, there were only a modest number cognitively normal older adults, or individuals with non-amnestic profiles or those with PreMCI (Impaired-Not MCI) who were p-tau positive. As such, findings among those with aMCI may not generalize to these particular groups. Finally, because many individuals with aMCI in our sample also showed impairments in non-memory domains, statistical power was limited to target the unique effects of memory vs. non-memory dysfunction on semantic intrusion errors.

The clinical implications of these findings are significant. Plasma p-tau_217_ (16) has emerged as the most sensitive and predictive biomarker of Alzheimer's disease. However, many individuals lack access to costly and time-intensive amyloid PET scans available only at major medical centers. Blood-based biomarkers offer an affordable and efficient means for early detection of amyloid and tau accumulation in the brain. Nevertheless, p-tau_217_ alone, without the inclusion of a brief cognitive challenge test such as the LASSI-L or similar assessments in community-based settings, may not hold sufficient clinical relevance. This underscores the need for further research to explore the link between blood biomarkers with sensitive cognitive testing to improve the early identification of persons at risk for AD/ADRD.

## Data Availability

The raw data supporting the conclusions of this article will be made available by the authors, without undue reservation.
